# Human menstrual blood-derived mesenchymal stem cells as a cellular vehicle for malignant glioma gene therapy

**DOI:** 10.18632/oncotarget.17621

**Published:** 2017-05-04

**Authors:** Xiao-Jun Wang, Bing-Yu Xiang, Ya-Hui Ding, Lu Chen, Hai Zou, Xiao-Zhou Mou, Charlie Xiang

**Affiliations:** ^1^ State Key Laboratory for Diagnosis and Treatment of Infectious Diseases, The First Affiliated Hospital, School of Medicine, Zhejiang University, Hangzhou 310003, China; ^2^ Department of Cardiology, Zhejiang Provincial People's Hospital, Hangzhou 310014, China; ^3^ Clinical Research Institute, Zhejiang Provincial People's Hospital, Hangzhou 310014, China; ^4^ Collaborative Innovation Center for Diagnosis and Treatment of Infectious Diseases, The First Affiliated Hospital, Zhejiang University School of Medicine, Hangzhou 310014, China; ^5^ People's Hospital of Hangzhou Medical College, Hangzhou 310014, China

**Keywords:** gene therapy, menstrual blood-derived mesenchymal stem cell (MenSC), malignant gliomas, tumor necrosis factor-related apoptosis-inducing ligand (TRAIL)

## Abstract

Despite many advances in conventional treatment strategies, there is no effective treatment modality for malignant gliomas. Gene therapy may offer a promising option for gliomas and several gene therapy approaches have shown anti-tumor efficiency in previous studies. Mesenchymal stem cell-based gene therapies, in which stem cells are genetically engineered to express therapeutic molecules, have shown tremendous potential because of their innate homing ability. In this study, human menstrual blood-derived MSCs (MenSC), a novel type of multipotential MSCs displays tropism for human malignant glioma when used as a gene delivery vehicle for therapeutics. Secretable trimeric TRAIL (stTRAIL) contains the receptor-binding domain of TRAIL, a death ligand that induces apoptosis in tumor cells. To overexpress stTRAIL, MenSCs were infected with efficient adenoviral serotype 35 vectors that had no influence on its broad multipotency and low immunophenotype. The modified MenSCs served as an excellent local drug delivery system for tumor site-specific targeted delivery and demonstrated therapeutic efficacy in an animal xenografts tumor model of U-87 MG cells. The MenSC-stTRAIL cells induced antitumor effects *in vitro* by significantly increasing apoptosis (*P* < 0.05). It also significantly reduced tumor burden *in vivo* (*P* < 0.05). The results showed that the proliferation of tumor cells was significantly reduced (*P* < 0.05). The MenSC, as a cellular delivery vehicle has a wide potential therapeutic role, which includes the treatment of tumors.

## INTRODUCTION

Malignant glioma is a highly aggressive primary intracranial tumor. The most common malignant glioma is glioblastoma multiforme (GBM, grade IV astrocytoma), for which median survival is only 9.9 to 15 months [1–3]. Despite many advances have been made in conventional treatment strategies, including surgery, radiotherapy, and chemotherapy, no effective treatment modality is available to treat gliomas. Gene therapy for gliomas is promising as it can be delivered *in situ* and selectively targets tumor cells.

Mesenchymal stem cell (MSC)-based gene therapies, wherein stem cells are genetically engineered to express therapeutic molecules, have shown tremendous potential in anticancer applications because of their innate ability to home onto tumors [4–7]. In addition to bone marrow (BM-MSCs), MSCs can be easily isolated from adipose tissue (AT-MSCs) and umbilical cords (UC-MSCs) and expanded *in vitro* [8–10]. However, it is significantly challenging to use these MSC tissue resources because isolating them generally requires extremely invasive procedures.

To circumvent these problems, a highly proliferative MSC was identified in menstrual blood by Meng et al. [11]. Human menstrual blood-derived mesenchymal stem cells (MenSCs) have been recognized as a novel source of stem cells [12]. MenSCs display stem cell-like phenotypic markers, a propensity for self-renewal, and high proliferative potential *in vitro*, in addition to the ability to differentiate into a diverse array of cell lineages [11–13]. They can be acquired without invasion, and their use avoids any ethical controversies. Moreover, our group and other researchers have reported that MenSC cells possess low immunogenicity, they can bestably expanded for 20 passages at least without genetic abnormal [14–17]. They therefore represent an ideal source for research into the potential applications of MSCs.

Tumor necrosis factor-related apoptosis-inducing ligand (TRAIL) is a type 2 transmembrane death ligand that induces apoptosis in target cells by activating the apoptosis pathway [18]. Structural analyses have demonstrated that secretable TRAIL (sTRAIL, amino acids 114–281) contains the protein's receptor-binding domain [19]. This is a promising candidate for cancer therapies because it can be used to selectively trigger apoptosis in cancer cells without damaging normal cells [20, 21].

In the present study, human MenSCs display tropism for human malignant gliomas. Additionally, MenSCs that were infected with an efficient adenoviral serotype 35 (Ad35) vector that overexpressed stTRAIL displayed significant antitumor effects *in vitro* and *in vivo*.

## RESULTS

### The infection efficiency of the adenovirus and the immunophenotype of MenSC-eGFPs

As human primary MSCs, BMSCs express only low levels of the coxsackie adenovirus receptor (CAR) [22]; therefore, they are relatively resistant to infection with wild-type adenoviral serotype 5 (Ad5). We first confirmed that MenSCs display similar expression levels of CAR (Supplementary Figure 1). It has been reported that CD46, the primary receptor for Ad35, is expressed on most human cells throughout the body and in MenSCs (Supplementary Figure 2) [23].

**Figure 1:**
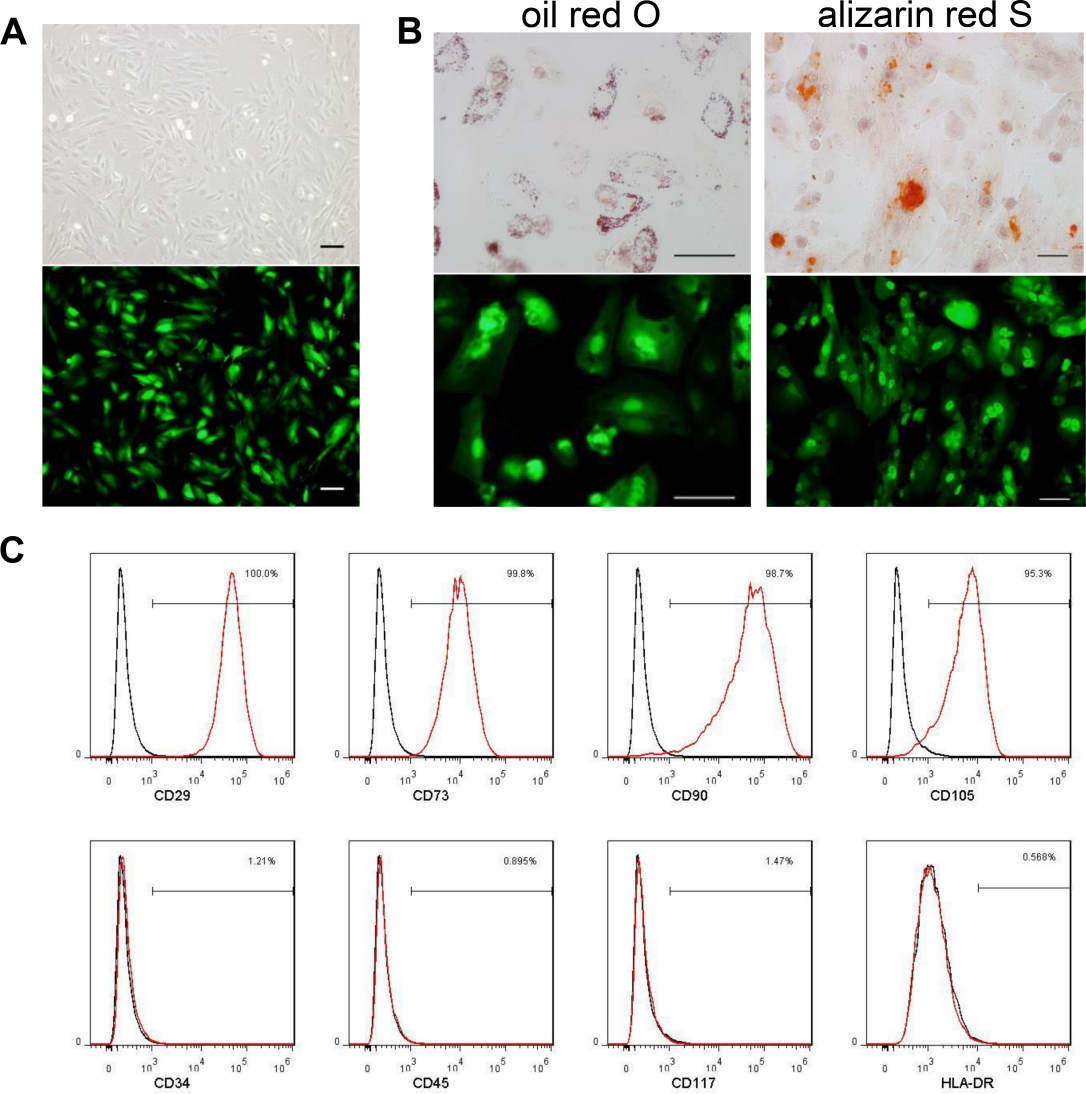
MenSCs were infected with Ad35-eGFP and Ad35-sTRAIL at a MOI of 20 The cells exhibited a spindle-shaped and fibroblast-like morphology (**A**). The MenSC-eGFP cells maintained their capability to undergo adipogenic and osteogenic differentiation. The differentiated cells are labeled with oil red O or alizarin red S (**B**). (Scale bar: 100 μm) The immunophenotypes of MenSC-sTRAIL cells were analyzed using surface markers, and no differences were found after adenovirus infection (**C**). MenSC-sTRAIL-expressing cells (red) and isotype controls (black).

MenSCs were infected with Ad35-eGFP and Ad35-sTRAIL at a MOI of 20. Similar to BMSCs, MenSC-eGFP cells exhibited a spindle-shaped, fibroblast-like morphology (Figure 1A). They had the ability to differentiate into adipogenic and osteogeniccells in differentiation induction medium (Figure 1B). The MSC-specific markers CD29, CD73, CD90, and CD105 were similarly expressed in MenSC-sTRAIL cells and control MenSCs (data not shown) [17], whereas the hematopoietic stem-cell markers CD34 and CD45 were not. The expression of CD117 and HLA-DR were also negative (Figure 1C). And the data of MenSC-eGFP was shown as (Supplementary Figure 3). These modifications to gene expression did not alter the biological properties of MenSCs.

### TRAIL protein detection in MenSC-sTRAIL cells and culture supernatants

**Figure 2:**
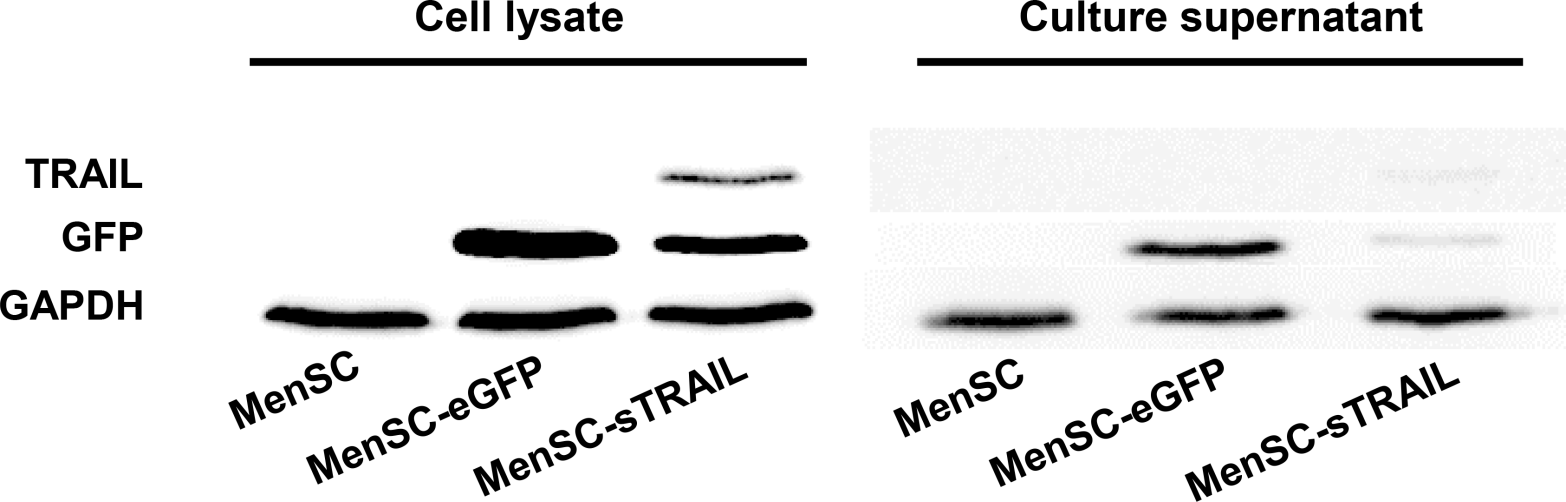
After cells were transfected with Ad35, the cell lysates and culture supernatants were obtained and evaluated using Western blot analysis Non-transfected MenSCs were used as the controls.

One day after infection, the resulting cell lysates and culture supernatants were evaluated using Western blot analysis. The results showed that there was clear sTRAIL protein expression in the MenSC-sTRAIL cells and culture supernatant (Figure 2). We also found that GFP was expressed at about half the level in MenSC-sTRAIL cells that was observed in MenSC-eGFP cells that were infected with Ad35 at the same MOI. These results confirm the observations that were made in the flow cytometry assays (Supplementary Figure 4).

### Migration of modified MenSCs toward gliomas *in vitro* and *in vivo*

**Figure 3:**
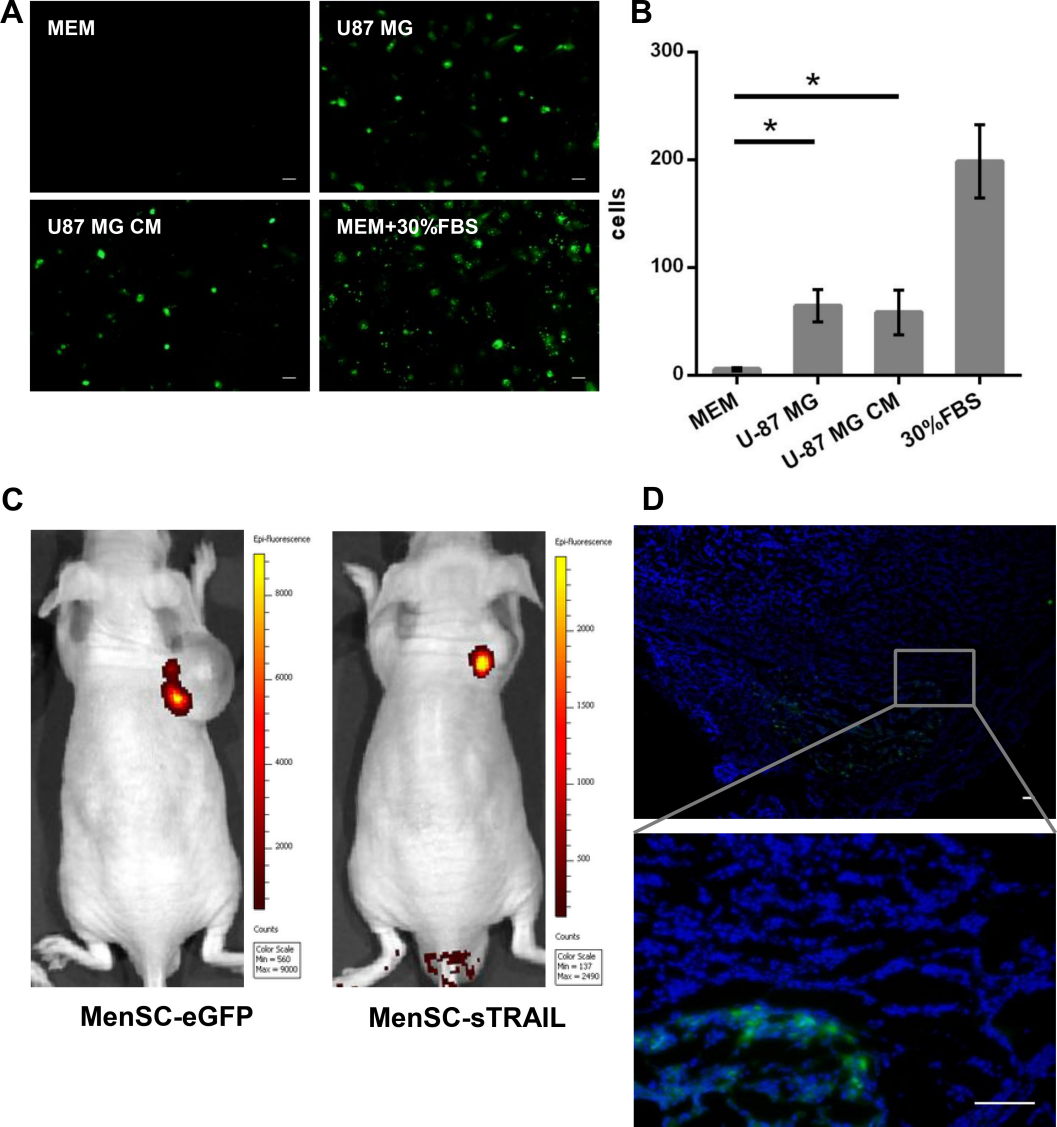
*In vitro* Transwell migration assays MenSC-eGFP cells were significantly (*p* < 0.05) attracted to the culture medium obtained from U-87 MG cells (**A, B**). The injected cells were identified using a small animal *in vivo* imaging system (**C**). Frozen tumor sections from the tumor of the mice injected with MenSC-eGFPs were counter stained with DAPI. The cells display green fluorescence and were observed both surrounding the tumor periphery and distributed throughout the tumor mass (**D**). (Scale bar: 100 μm)

To test the ability of gene-modified MenSCs to migrate toward specific attractants, we performed *in vitro* assays using Transwell plates. While a few MenSC-eGFP cells were observed to migrate toward serum-free medium, cell migration was significantly (*P* < 0.05) increased by U-87 MG or its culture supernatants (Figure 3A, 3B). These results indicate that U-87 MG cells are capable of stimulating the migration of MenSCs and that the migratory capacity of these cells was not affected by adenoviral transduction.

To evaluate the effect of U-87 MG xenograftson the tumor-influenced migration of MenSC-sTRAIL cells, mice received 1 × 10^6^ MenSC-GFP or MenSC-sTRAIL cells via tail vein injection once per week. As shown in Figure 3C, the injected cells were identified using a small animal *in vivo* imaging system. We found that there was stronger green fluorescent signal in the tumors of the mice injected with MenSC-eGFP cells than in those injected with MenSC-sTRAIL cells. In the frozen tumor sections from tumor in Men-eGFP treating group, cells expressing green fluorescence both surrounded the tumor periphery and were distributed throughout the tumor mass (Figure 3D). The section from Men-sTRAIL group was displayed as a (Supplementary Figure 5).

### MenSC-sTRAIL inhibits proliferation and induces apoptosis *in vitro*

**Figure 4:**
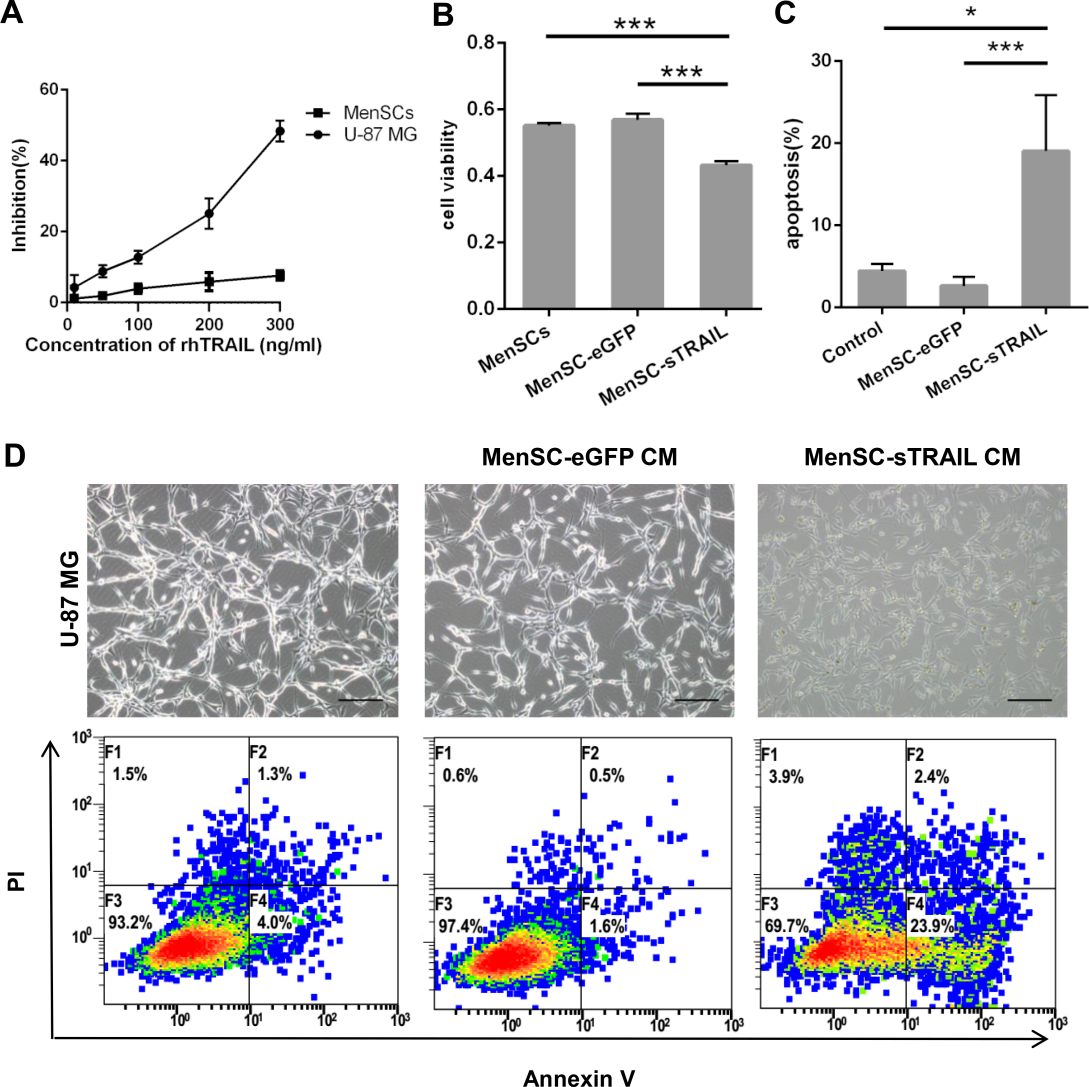
Confirmation that sTRAIL effectively induced tumor cell death U-87 MG cells showed lower than 60% viability when rhTRAIL levels were increased to 300 ng/ml (IC50 = 339.4 ng/ml) (**A**). The supernatants of MenSC-sTRAIL cells were transferred to cultures of U-87 MG cells, resulting in decreased viability in the tumor cells (*p* < 0.01) (**B**) and induced a significantly higher rate of apoptosis in tumor cells (*p* < 0.05) (**C**). The CM of MenSC-sTRAIL cells lowered cell densities and adherence in U-87 MG cultures. However, the morphologies of the cells in the other groups were not significantly altered (**D**). (Scale bar: 100 μm).

We incubated U-87 MG and MenSCs with increasing concentrations of rhTRAIL. We observed no significant decrease in cell survival in the treated MenSCs, but the treated U-87 MG cells showed a 60% decrease in viability when rhTRAIL levels were increased to 300 ng/ml (Figure 4A).

To determine the bioactivity of this secreted protein, we analyzed U-87 MG cell viability and apoptosis after cells were incubated with MenSC-sTRAIL culture supernatants. After 24 h of exposure, the U-87 MG cells showed a decrease in viability (*P* < 0.01) (Figure 4B) and a more than 20% increase in apoptosis (Figure 4C). These results were significantly different (*P* < 0.05) than the results observed when cells were exposed to MenSC-eGFP CM or conditional medium (Figure 4C). However, while the cell morphology, density and adherence of the U-87 MG cells decreased after exposure, these characteristics were not altered in the control cells (Figure 4D).

### MenSC-sTRAIL reduce subcutaneous xenografts tumor growth

**Figure 5:**
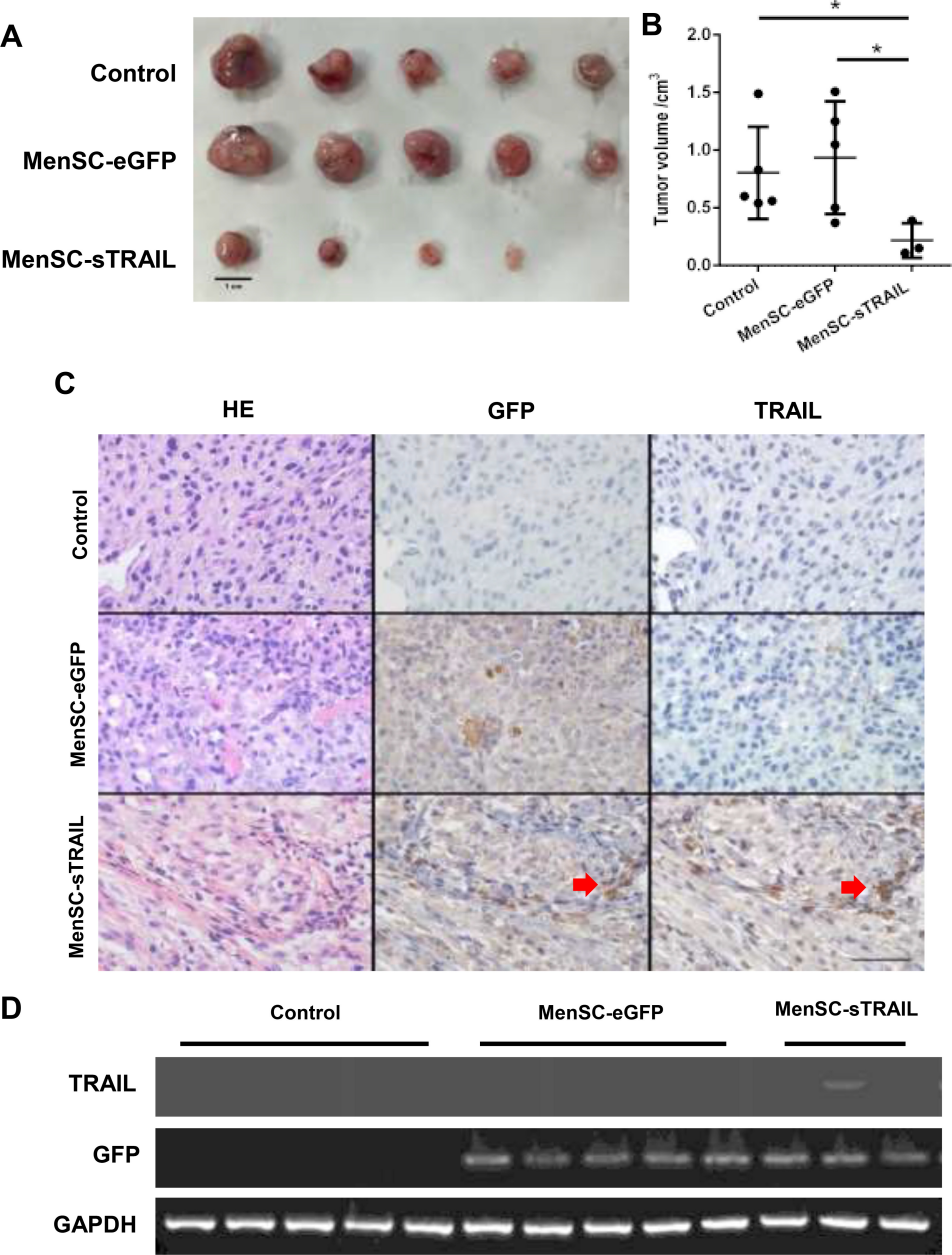
After three tail vein injections, tumor size was significantly lower (*p* < 0.05) in the mice injected with MenSC-sTRAIL (**A, B**) (Scale bar: 1 cm). The expression levels of the TRAIL and GFP proteins are shown in a tumor tissue section. Both GFP and TRAIL were expressed at the same location as indicated (**C**) (Scale bar: 100 μm). The mRNA expression level of TRAIL is shown (**D**).

**Figure 6:**
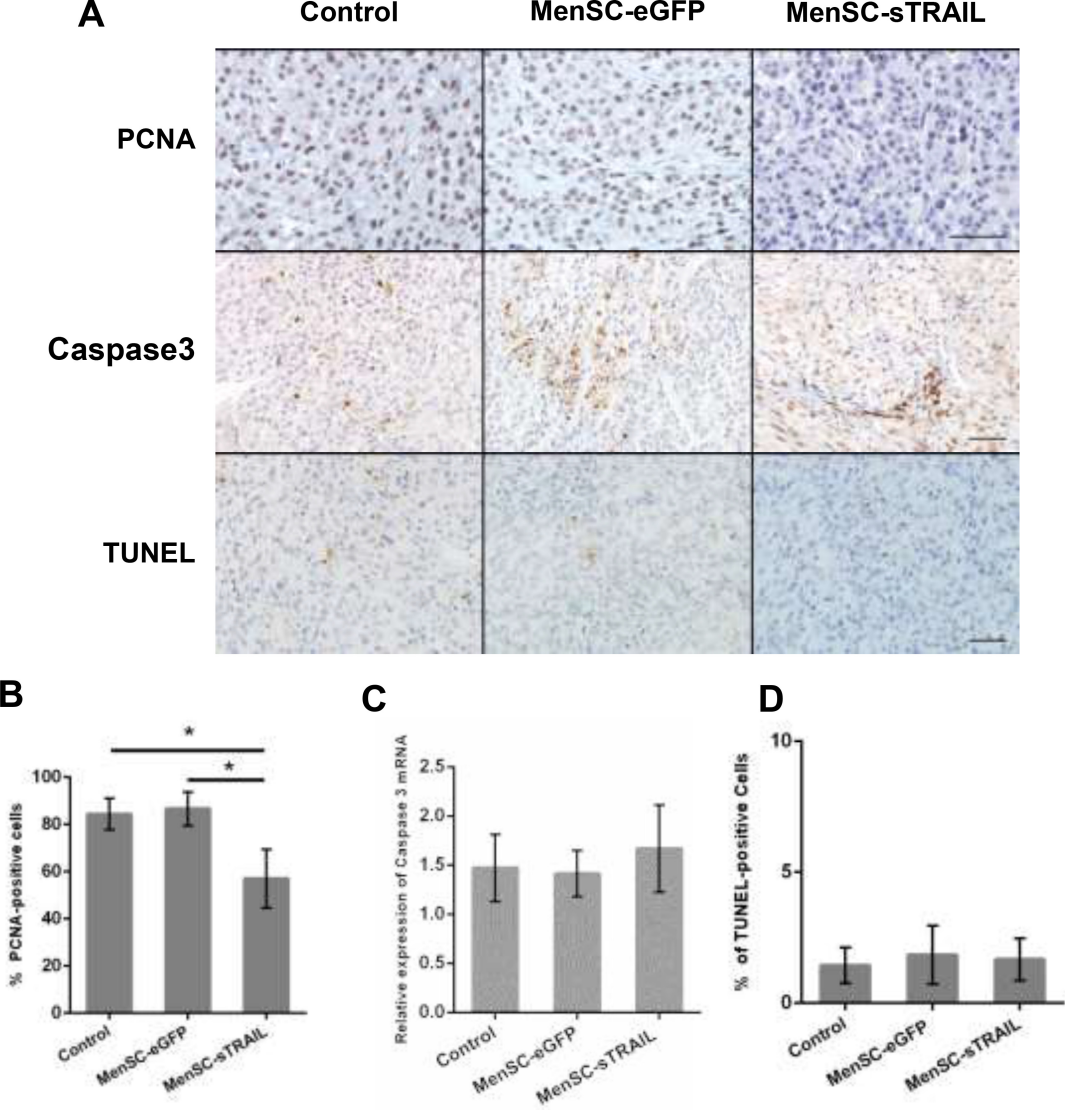
We evaluated the level of PCNA and cleaved Caspase 3 protein expression (**A**) (Scale bar: 100 μm). The results showed that sTRAIL reduced tumor proliferation (*p* < 0.05) (**B**) but did not induce apoptosis. Cleaved Caspase 3 was not significantly upregulated at the mRNA level (**C**). There was no difference in the number of TUNEL-positive cells across the three groups (A, **D**).

We next sought to determine whether MenSC-sTRAIL cells also have anti-tumor activity *in vivo*. After a series of three tail vein injections, tumor size was significantly reduced (*P* < 0.05) in mice injected with MenSC-sTRAIL (Figure 5A, 5B). In two out of five mice, the tumor vanished. As shown in Figure 6A, the smallest tumor was observed in a MenSC-sTRAIL-injected mouse, and H&E stating section was confirmed to be composed of fibro tissue by two pathologists. A mouse that was injected with MenSC-eGFP had the largest tumor volume. However, there was no significant difference in tumor volume between the control and MenSC-eGFP-injected mice (Figure 5B).

### Expression of TRAIL *in vivo*

Upon detecting the expression of TRAIL, we found that both GFP and TRAIL were expressed at the same location as shown in Figure 5C, confirming the activation of MenSC-sTRAIL cells that had migrated to the tumor periphery and become distributed throughout the tumor mass. Without TRAIL expression, GFP expression was higher in tumors in the MenSC-eGFP group. We also extracted total RNA from the tumor tissues and then detected TRAIL and GFP gene expression at the mRNA level, as shown in Figure 5D.

### TRAIL induced growth inhibition and apoptosis *in vivo*

To determine the mechanism by which TRAIL induces growth inhibition, we evaluated the level of PCNA expression (Figure 6A). The results showed that there were significantly fewer PCNA-positive cells in the tumors exposed to MenSC-sTRAIL than in the other two groups (*P* < 0.05) (Figure 6B).

We next detected cleaved caspase 3 expression levels (Figure 6A). We initially observed that the protein expression level was higher, but the data obtained from RT-PCR showed that there was no significant difference in its expression at the mRNA level (Figure 6C). We than stained sections using a TUNEL assay kit to detect apoptotic activity (Figure 6A); the results showed that there was no significant difference across the three evaluated groups (Figure 6D).

## DISCUSSION

Attempts to use MSCs as cellular delivery vehicles for tumor therapies have therefore combined their homing ability with the relative ease of expanding their numbers, the lack of ethical concerns and their lack of immunogenicity. However, BM-MSCs, AT-MSCs and UC-MSCs are limited in more widely use for invasiveness of extraction, restricted differentiation potential, or in some cases, a limited proliferative capacity. Compared with them, our colleagues had successfully isolated and identified a population of MSCs from human menstrual blood (MenSCs) [14, 16]. These cells exhibit a higher proliferation rate and can be obtained through a simple, safe, painless procedure that is not ethically controversial. A single definite marker of the *in vivo* MSCs had yet to be identified. According to Pittenger's report, human MSC is characterized by the presence of particular surface markers, including CD29, CD44, CD71, CD90, and CD105, and by the absence of marker of leukocytes and cells of hematopoietic lineage, including CD14, CD34, and CD45 [10].

Vectors of Ad35 were constructed to express the sTRAIL gene, and these vectors were used to modify MenSCs. These gene modifications did not alter the morphology or the immunophenotype of the MenSCs.

Several studies have confirmed that MSCs migrate toward human gliomas *in vivo*, [5, 24] and transduced MSCs retain a migratory ability that is similar to the pattern observed in untransduced MSCs [25]. Our colleague confirmed MenSCs are chemoattracted toward SDF-1α, which is highly expressed by U-87 MG cells, because they have cell surface expression of the CXCR4 and CXCR5 receptors (data not shown) that are similar to the levels reported on common MSCs [26]. In this study, we confirmed that infected MenSCs retain their migratory capacity. To confirm whether the tumor cells could produce enough cytokines or chemokines to attract tropic cells, we need to detect the SDF-1α expression *in vivo* model. These data suggest that MenSCs are an excellent cellular local drug delivery system for tumor site-specific targeted delivery that may achieve a high degree of therapeutic efficacy. They therefore represent an ideal source for research and clinical work into the potential applications of MSCs. But clinical applications are still challenging and more work needs to be done.

Intracranial GBM tumor models used in preclinical research studies have indicate that murine U-87 MG models closely reproduce the histopathological features of human GBM, even though the tumor cells were subcutaneously injected into the right flank in some other studies [27]. But in this study, the green fluorescent signal was hard to be detected using the Small animal *in vivo* imaging system, if the MenSC-eGFP and MenSC-sTRAIL cells migrated toward the U-87 MG cells intracranial injected. To confirm the homing ability *in vivo* of gene modified MenSCs, we firstly implanted the tumor cells subcutaneously. For further study, we are trying to constructed Luciferase expressing MenSCs, so the migration of Fluc-MenSC to intracranial U-87 glioma tumor could be monitored using the Small animal *in vivo* imaging system. We used subcutaneous injection of tumor cells to model this disease so that we could avoid the trauma that is caused by the use of skull punching and the cerebral lesions cause by the used of multiple injections.

Studies of gene therapy that have used MSCs have shown promise, but the blood brain barrier (BBB) remains an obstacle that essentially restricts therapeutic drugs from entering the brain. Delivering anticancer drugs to tumor sites is therefore a major challenge for treatment saimed at brain cancers [28]. Different kinds of stem cells have been inserted via intracerebral or carotid artery injection [29, 30]. However, stem cells have been confirmed to migrate towards damaged brain tissues after intravenous injection in many studies [31, 32]. In Zhu's study, labeled human umbilical cord MSCs were detected in the damaged periventricular white matter of a model animal [33]. Matsushita, Kibayashi [34] developed an *in vitro* culture system that consisted of brain microvascular endothelial cells (BMECs) and BM-MSCs cultured in Transwell inserts, and their data showed that the MSCs transmigrated across the BMEC monolayers. To remove the obstacle presented by the BBB to drug delivery, exosomes, which are small membrane-bound vesicles that are secreted by a multitude of cell types, have been successfully used to deliver therapeutic agents to the brain in rodent models [35, 36], Yang, Martin used brain endothelial cell-derived exosomes as a carrier for an anticancer drug that was delivered to the brain to treat brain cancer [37]. Our colleague successfully isolated exosomes from MenSCs [38].

Although human MSCs are filtered by the lungs, the systemic administration of MSCs via tail vein injection remains the most commonly used method. Our group had reported that the MenSCs could survive and be detected after more than 2 weeks in the normal immune mice model after the tail vein injections [14, 17]. In our study, the therapeutic effects of MenSC-sTRAILcells on our tumor models were not conspicuously limited by the administration route. Figure 6C shows a section of tumor tissue from a mouse that was treated with MenSC-sTRAIL cells. This section demonstrates that cells in this tumor were both GFP (+) and TRAIL (+). The GFP-and TRAIL-positive granules were scattered throughout the tissue and distributed mostly around the tumor site, verifying that the interaction between MenSCs and tumor cell was influenced by a paracrine mechanism.

MSCs have been used in cancer therapies to deliver many types of drugs. Stably transfected BMSCs were able to penetrate tumor sites and act as a reservoir that slowly released the therapeutic protein TRAIL [39]. Kim, Lim [40] also reported on the therapeutic efficacy of TRAIL-secreting umbilical cord blood-derived MSCs that were transduced with an adenovirus. The recombinant human TRAIL protein we used can not be used clinically because of its short half-life and pronounced toxic effects on non-malignant cells in the body. Using MSCs to deliver a TRAIL peptide would solve both of these problems. Once there, they locally express the protein, greatly increasing the TRAIL concentration within the tumor, resulting in long-lasting effects and significantly lowering its systemic toxicity. Other studies have provided evidence showing that MSCs have the ability to actively seek out metastatic sites that are far removed from the primary tumor site [41]. Before gene transduction, we confirmed that the sTRAIL protein would induce apoptosis only in tumor cells and not in MenSCs. We next detected the sTRAIL expression level in the culture medium because it might have directly induced apoptosis in the tumor cells. As shown in the results, the 15–30% TRAIL killing of U-87 was induced by MenSC-sTRAIL culture supernatants. TRAIL was faintly expressed. However, if we co-culture the MenSC-sTRAIL and U-87 cells in a transwell, the stably transfected MenSC-sTRAIL were able to act as a reservoir that slowly released the therapeutic protein. We may observe a higher killing of tumor cells.

Interactions between MSCs and tumor cells have become a focus of research based on the possibility that MSCs may have tumor-promoting properties [42, 43]. Our data support this notion: our co-culture experiment showed that MenSC-eGFP CM resulted in a lower rate of apoptosis than was observed in cells exposed to control CM, and in our *in vivo* mouse tumor model, the largest tumor was observed in a mouse that was injected with MenSC-eGFP cells. The *in vivo* therapeutic efficacy of these cells confirmed that treatment with MenSC-sTRAIL resulted in significantly lower tumor volumes. Moreover, two out of the five mice were tumor-free at the end of the experiment. To achieve better results, we may need to use these cells in combination with other chemotherapeutic drugs.

PCNA expression levels were detected to explore the mechanism. We found that significantly fewer PCNA-positive cells were observed in tumors exposed to sTRAIL expression than in the other groups. However, there was no difference in the rate of apoptosis in tissues between the three groups, and this was unexpected. We next detected the mRNA and protein expression levels of cleaved Caspase 3, a component of the TNF-induced apoptosis pathway, but there was no significant difference across the groups. Some studies have shown that there are interactions between apoptosis and cell survival pathways. NF-κB is activated, and the activation of NF-κB blocked the activation of caspase-8, which is the activator of caspase-3 in the TRAIL-induced apoptosis pathway [44, 45]. More work is needed to determine the interactions between these pathways.

## MATERIALS AND METHODS

### Cell Isolation and culture

Menstrual blood samples were collected from healthy female subjects aged from 25 to 40 years. MenSCs were isolated and cultured as previously described [11, 14, 17]. All isolation and culture procedures were performed with the consent of the donors and approved by the Ethics Committee of the First Affiliated Hospital, Zhejiang University, China. First, blood samples were collected using a Diva cup (Kitchener, Canada). The samples were centrifuged, and mononuclear cells were separated using density gradient centrifugation in Ficoll-Paque (Fisher Scientific, USA). The interlayer cells were collected and cultured in Chang Medium to obtain adherent cells. All MenSCs used in these experiments were at passage 6 to 8.

U-87 MG cells were originally obtained from American Type Culture Collection (ATCC) and cultured in Minimum Essential Medium Eagle (MEM; Corning, USA) supplemented with 10% fetal bovine serum (FBS; Gibco, USA) and 1% penicillin/streptomycin. All cells were incubated at 37°C in a 5% CO_2_ atmosphere in an incubator.

### Adenoviral vectors construction and infection

**Figure 7:**
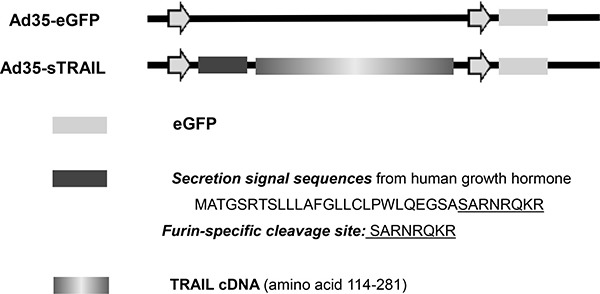
Construction of sTRAIL expression vector The secretion signal sequences (N-terminal 26 amino acids of human growth homone) including Furin-specific cleavage site, and sTRAIL (amino acids 114–281) sequence was inserted into the Ad35-eGFP vector.

We used Ad35 as the gene expression vector. The enhanced green fluorescent protein (eGFP) and sTRAIL genes were inserted into the Ad35 vector. Ad35-eGFP served as the vehicle vector (Figure 7). The furin cleavage site is a specific protease cleavage sequence that is recognized by Furin. The furin cleavage site will eliminate secretion signal sequence from the final active TRAIL in the process of protein secretion [46].

MenSCs were infected with Ad35 at a multiplicity of infection (MOI) of 5 to 50 for at least 6 hours and then washed with phosphate-buffered saline (PBS; Corning, USA) to remove unincorporated Ad. The lowest volume of MOI at which the GFP-positive cells were more than 90% was used as the final MOI volume.

### Phenotypic characterization and induced differentiation of modified MenSCs

To identify modified MenSCs, the cell surface markers were detected using fluorescence-activated cell sorting (FACS). A total of 5 × 10^5^ cells were collected and incubated with antibodies to various cell surface markers, including CD29, CD34, CD45, CD73, CD90, CD105, CD117, and HLA-DR. The stained cells were re-suspended and analyzed using a FC500 flow cytometer (Beckman, USA). IgG1 and IgG2a served as the isotype controls.

MenSC-eGFP cells were plated at a density of 2 × 10^4^ cells/cm^2^ on 6-well plates and cultured in special conditional osteogenic or adipogenic medium (Cyagen, China). Osteogenic differentiation was then evaluated using Alizarin Red S staining, and adipogenesis was confirmed using oil red O.

### Experimental animal tumor model and cells transplantation

Male nude mice (6–8 weeks old, 20–25 g) were purchased from Sippr-BK Laboratory Animal Corporation (Shanghai, China) and housed under standard conditions at the Laboratory Animal Center of Zhejiang University (Hangzhou, China). All animal experiments were approved by the Ethics Committee of the First Affiliated Hospital, Zhejiang University. For the *in vivo* experiments, 1 × 10^6^ U-87 cells were subcutaneously injected at a dorsal site. When nodules under the skin were visible, the mice were randomly divided into three groups. The mice in the control group received 500 μL PBS via tail vein injection at day 1, day 8, day 15, while the experimental groups received 1 × 10^6^ of either MenSC-eGFP cells or MenSC-sTRAIL cells. After three tail vein injections, each mouse received 3 × 10^6^ cells totally before the tumors were harvested at day 21.

### *In vitro* and *in vivo* migration studies

The migratory ability of MenSC-eGFP cells was determined using Transwell inserts (Corning, USA). U-87MG cells (1 × 10^6^) were incubated in serum-free medium for 24 h, and the resulting conditioned medium was used as the chemoattractant. MenSC-eGFP cells (1 × 10^4^ cells) were plated in Transwell inserts. Serum-free medium or MEM medium with 30% FBS was placed in the lower wells and used as controls. After 48 hours, the cells on the upper sides of the filters were wiped off, and the cells that had migrated to the lower sides were counted under a fluorescence microscope (IX83, Olympus, Japan).

For the *in vivo* migration study, the injected cells were analyzed using an IVIS Spectrum 3D Small animal *in vivo* imaging system (PerkinElmer, USA). Frozen tumor sections were counterstained with4′, 6-diamidino-2-phenylindol (DAPI; Sigma, USA) (1 μg/ml) to detect migration of the MenSC-eGFP and MenSC-sTRAIL cells under a fluorescence microscope.

### Western blotting

After cells were infected, the culture supernatants were centrifuged and concentrated. The cells were then lysed. Proteins were separated and then transferred onto polyvinylidenedifluoride (PVDF) membranes (Millipore, USA). After blocking, the membranes were incubated with TRAIL antibody (Abcam, UK), GAPDH antibody (GeneTex, USA), or GFP antibody (GeneTex, USA) overnight at 4°C. After the membranes were incubated with horseradish peroxidase-conjugated secondary antibody (Bio-Rad, USA), the bands were detected using Western ECL Blotting Substrate (Bio-Rad, USA).

### Reverse transcriptase polymerase chain reactions and real-time PCR

The expression of transgenes in transduced cells was confirmed using reverse transcription-polymerase chain reaction (RT-PCR). Total RNA was extracted from tissues using Trizol Reagent (Invitrogen, USA) and was then reverse transcribed using PrimeScript Reverse Transcriptase (TaKaRa, Japan). PCR products were resolved on gelsthat were stained using ethidium bromide (EB) and visualized using a UV transilluminator. Real-time PCR was performed using a Bio-Rad CFX96 touch q-PCR system (Bio-Rad, USA) with SYBR Green Premix Ex Taq (TaKaRa, Japan). The relative expression of each gene was normalized to the expression level of endogenous GAPDH. The primer sequences that were used in these reactions are shown in Supplementary Table 1. Relative gene expression levels were calculated using the 2^-ΔΔCT^ method.

### *In vitro* tumor cell viability and apoptosis

Cell viability was measured using a Cell Counting Kit-8 (CCK-8; Dojindo, Japan). U-87 MG (5 × 10^3^/well), MenSCs (2 × 10^3^/well) were seeded in 96-well plates to measure the cytotoxicity that was induced by various concentrations of recombinant human TRAIL (rhTRAIL, Val114-Gly281) (R&D Systems, USA).

To measure the level of cytotoxicity that was induced by sTRAIL, the MenSC-sTRAIL cells were cultured without FBS for 24 h. After centrifugation, the culture medium was then transferred to adherent U-87 MG cells. We used MenSC culture medium and the MenSC-eGFP culture supernatant as the controls. U-87 MG cells from each of the three groups were then cultured for an additional 24 h.

U-87 MG cells were seeded in 12-well plates and cultured in the supernatants obtained from MenSC-sTRAIL cells for one day and photographed. Apoptosis was evaluated using a fluorescein isothiocyanate Annexin V Apoptosis Detection Kit (BD Biosciences, USA). The results were analyzed using flow cytometry (FC500MCL; Beckman Coulter, CA, USA).

### Evaluation of tumor size and histologic analysis

Tumors were harvested at day 21 after transplantation. Tumor volume was calculated as “length × width^2^/2” [47]. The tumor tissues were then fixed in formalin, embedded in paraffin and cut into sections (5 μm thick). The sections were deparaffinized, rehydrated, and stained with hematoxylin and eosin (H&E) for histology.

### Immunohistochemistry and *in vivo* apoptosis assays

For immunohistochemistry (IHC), the sections were heated in citrate buffer (0.02 M, pH 5.8) for antigen retrieval. The sections were incubated with diluted primary antibodies against TRAIL (1:100; Santa Cruz, USA), GFP (1:500), PCNA (Proliferating Cell Nuclear Antigen, 1:100; BBI, Canada) and cleaved caspase 3 (1:1000; Abcam, UK) at 4°C overnight. The primary antibodies were then detected using horseradish peroxidase-conjugated secondary antibodies (Abcam, UK). Peroxidase activity was visualized by exposing the tissues to diaminobenzidinetetrahydrochloride solution (DAB). The sections were then washed, counterstained with hematoxylin for one minute, mounted, and observed under a light field microscope. To detect apoptotic activity, sections were stained using a terminal deoxyribonucleotidyltransferase-mediated dUTP nick endlabeling (TUNEL) assay kit (Roche, Swiss).

### Statistical analyses

All statistical data were analyzed using one-way analysis of variance (ANOVA) with SPSS 16.0 software. The results are expressed as the mean ± standard deviation. A *P*-value < 0.05 was considered to indicate statistically significant.

## CONCLUSIONS

We have shown that menstrual blood-derived mesenchymal stem cells migrate toward gliomas, and we developed an efficient adenoviral serotype 35 transduction system for MenSCs in which MenSCs expressing sTRAIL were demonstrated to have excellent efficacy as a local drug delivery system that specifically targeted sTRAIL delivery to tumor sites in a glioma tumor model.

## SUPPLEMENTARY MATERIALS FIGURES AND TABLE


