# Supplementary material for: Human menstrual blood-derived mesenchymal stem cells as a cellular vehicle for malignant glioma gene therapy
Source: Oncotarget. 2017 May 4;8(35):58309–21. doi: 10.18632/oncotarget.17621 (PMC5601654; doi:10.18632/oncotarget.17621)
Supplement: Supplementary file 1 [file oncotarget-08-58309-s001.pdf]

Human menstrual blood-derived mesenchymal stem cells as a cellular vehicle for malignant glioma gene therapy

SUPPLEMENTARY MATERIALS

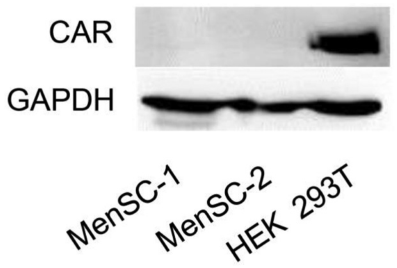

Supplementary Figure 1: We confirmed that MenSCs display negative expression of CAR with HEK293T cells as a control.

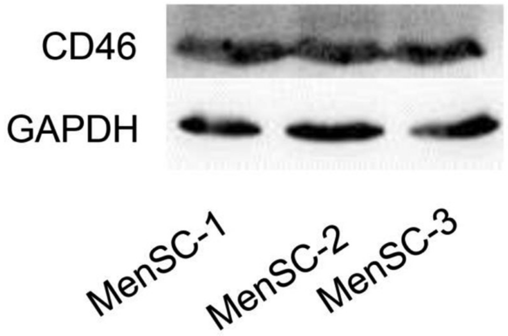

Supplementary Figure 2: CD46, the primary receptor for Ad35, is expressed in MenSCs.

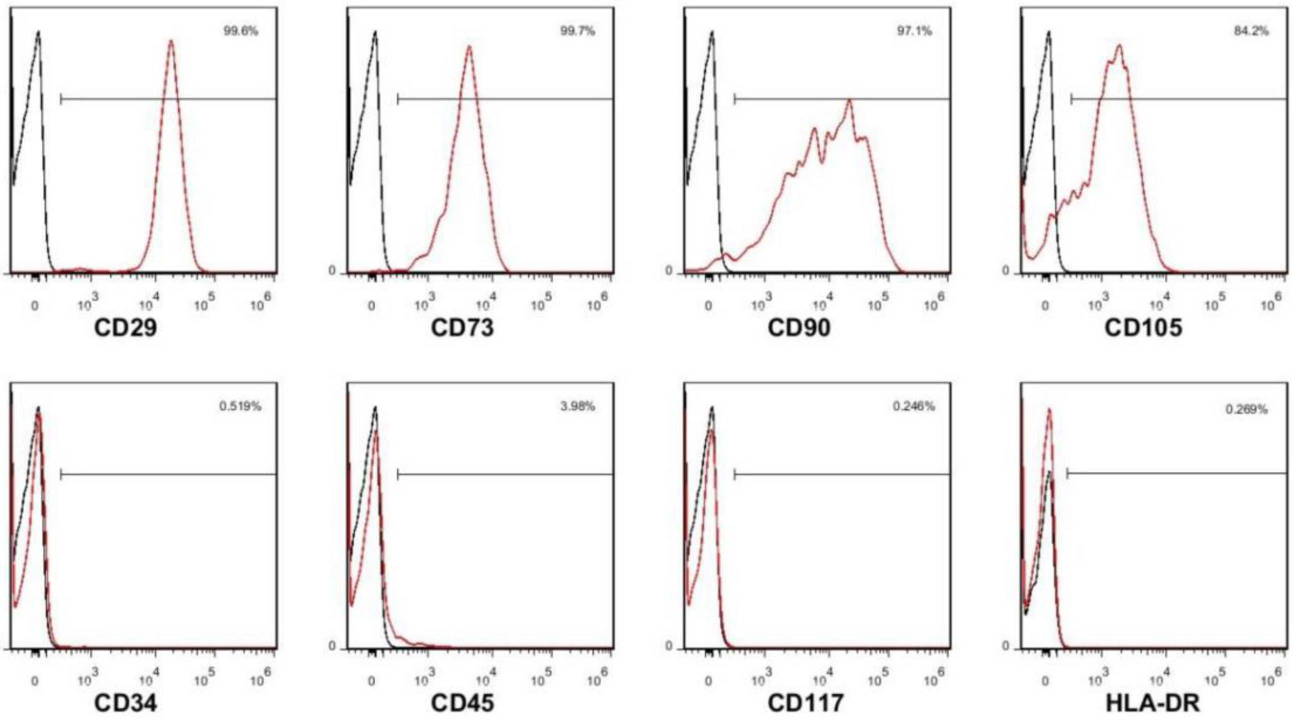

Supplementary Figure 3: The characterization of the immunophenotype of the genetically modified MenSC -eGFP cells. MenSC-eGFP cells (red) and isotype controls (black).

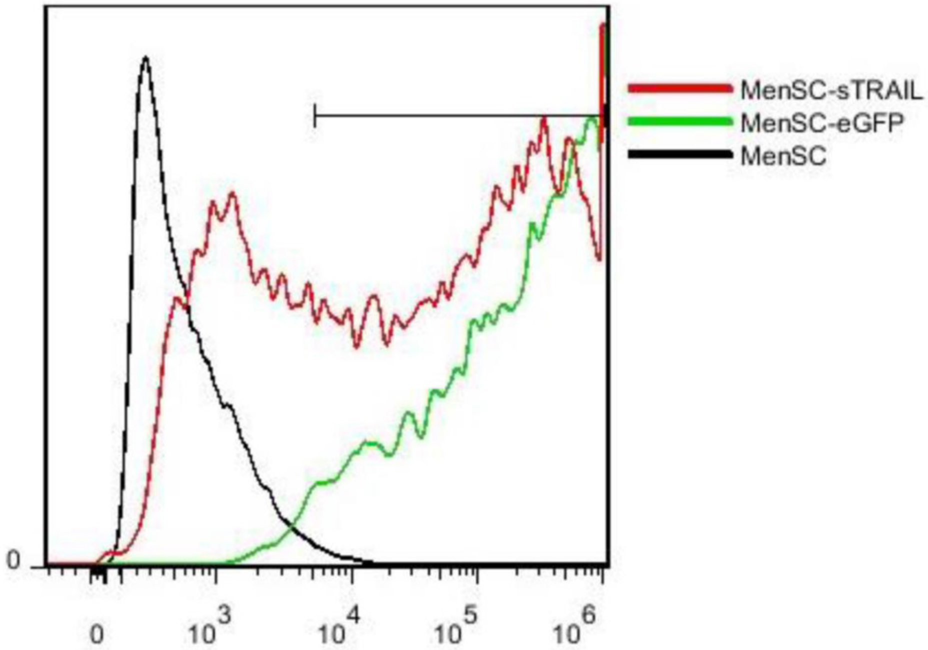

Supplementary Figure 4: In the flow cytometry assays, GFP was expressed at approximately half the level in MenSC-sTRAIL cells as that observed in MenSC-eGFP cells that were infected with Ad35 at the same MOI.

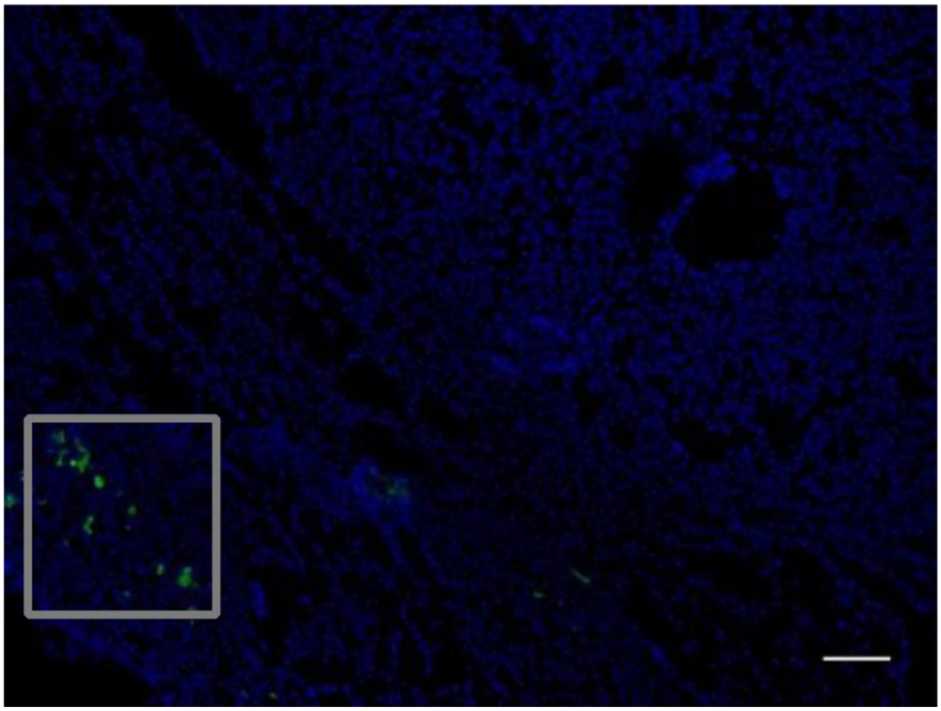

Supplementary Figure 5: The frozen tumor sections from tumor in Men-sTRAIL treating group. (Scale bar: 100 μm).

Supplementary Table 1: Primer sequences in PCR

Gene		Primers (5′–3′)
**TRAIL**	**F**	AGAGGAAGAAGCAACACAT
	**R**	CCTTGATAGATGGAATAGAGTC
**GFP**	**F**	GCCGACAAGCAGAAGAA
	**R**	AACTCCAGCAGGACCAT
**Caspase 3**	**F**	TACCGGTGGAGGCTGACT
	**R**	GCTCGAAAGGGACTGGAT
**GAPDH**	**F**	AATGGATTTGGACGCATTGGT
	**R**	TTTGCACTGGTACGTGTTGAT
